# Development of a simple algorithm to detect big air jumps and jumps during skiing

**DOI:** 10.1371/journal.pone.0307255

**Published:** 2024-07-18

**Authors:** Stefan Kranzinger, Christina Kranzinger, Aaron Martinez Alvarez, Thomas Stöggl

**Affiliations:** 1 Salzburg Research Forschungsgesellschaft mbH, Human Motion Analytics, Salzburg, Austria; 2 Red Bull Athlete Performance Center Los Angeles, Santa Monica, CA, United States of America; 3 Red Bull Athlete Performance Center Salzburg, Salzburg, Austria; Sunway University, MALAYSIA

## Abstract

Jumping is an important task in skiing, snowboarding, ski jumping, figure skating, volleyball and many other sports. In these examples, jumping tasks are a performance criterion, and therefore detailed insight into them is important for athletes and coaches. Therefore, this paper aims to introduce a simple and easy-to-implement jump detection algorithm for skiing using acceleration data from inertial measurement units attached to ski boots. The algorithm uses the average of the absolute vertical accelerations of the two boots. We provide results for different parameter settings of the algorithm and two types of jumps: *Big Air jumps* and *jumps during skiing*. The latter are divided into *small* (time of flight < 500 ms) and *medium* (time of flight ≥ 500 ms) jumps. The algorithm detects 100% of *Big Air*, 94% of *medium* and 44% of *small jumps*. In addition, the settings with the highest detection rates also have the highest number of overdetected jumps. To resolve this conflict, a penalty-adjusted score that considers the number of overdetected jumps in the final performance analysis is proposed.

## Introduction

Jumping is an important task and a performance criterion in many sports. Therefore, detailed knowledge about jumping is important for athletes and coaches. Besides standard metrics of jump performance as jump duration, jump height and jump distance, the jump analysis can be used to infer the total load on the body from jumps and landings, or it could contribute to the introduction of gamification applications in the amateur field. In the case of freeriding, it can be applied to show how long and far a jump was and, as a consequence, the trajectory of a jump could also be concluded. Therefore, the correct detection of jumps can be a powerful tool to help athletes and coaches control load and establish training targets.

The methodology for jump detection is based on tape measures [1], laser based distance calculation [2], force plates [1, 3], pressure sensor and insoles [4] or video analysis [5]. However the feasibility of these measures is not well adaptable to in-field measures. Therefore, minimal invasive on-body sensor technology might be a tool to be used in these settings. Inertial measurement units (IMUs) are used for sport performance evaluation and kinematic analysis [6, 7] in various sport disciplines e.g. from indoor sports (e.g. volleyball [8–10], combat sports [11] or figure skating [12, 13]) to outdoor or winter sports (e.g. snowboarding [14], golf [15], ski jumping [16] or alpine skiing [17, 18]). For alpine skiing [18] a scoring algorithm was developed to quantify the quality of movement with the potential to evaluate long-term performance and training management. Therefore, IMUs are well suited for monitoring athletes in various sports and could serve as a tool for analysis and more efficient training programming.

In existing literature authors already developed algorithms to estimate vertical jump heights of different jump types, such as squat jumps [19], countermovement jumps [19–21] or plyometric jumps [20]. The IMUs used in existing literature are either attached directly on the body or mounted on devices. Sadi and Klukas [22] proposed an algorithm for snow sports jump detection using a head-mounted Micro-Electro-Mechanical System (MEMS) IMU. The algorithm is based on two methods developed by the authors, which are *Windowed Mean Canceled Multiplication* and *Preceding and Following Acceleration Difference*. Their results showed that 92% of jumps during snowboarding, including ollie, step-up, cliff drop, and standard jump, were detected correctly. Lee et al. [23] detected jumps based on a threshold method for skiing and snowboarding. The authors used IMUs mounted on the helmet and additionally a MEMS barometric pressure sensor to detect jumps during skiing and snowboarding. However, according to Lee et al. [23] the head is not the perfect place due to head movements. Roberts-Thomson et al. [24] presented a fuzzy logic-based algorithm for jump detection in sports using accelerometer data of IMUs. The algorithm was applied to snowboarding and ski jumping, with IMUs mounted directly on the snowboards and skis, and successfully detected 92% and 100% of the jumps respectively. However, alongside traditional skiing and snowboarding, freestyle skiing is becoming more popular since the introduction of slope style as an Olympic discipline [25]. Therefore, it is crucial for coaches and athletes to distinguish the detection performance for different types of jumps in skiing. In addition, the implementation of a fuzzy logic-based algorithm (see [24]) could be challenging and thus be associated with limitations for users. Consequently, this work aims to develop an algorithm based on IMU acceleration data to detect jumps under different skiing conditions such as *Big Air jumps* and *jumps during skiing*. One advantage over other studies is that the proposed algorithm uses a simple and easy-to-implement structure. To overcome the problem of accounting for unwanted movements, the IMUs are attached to the skier’s boots, which should allow for stable data recording. In addition, we propose a method to resolve the trade-off between detected and overdetected jumps by introducing a penalty-adjusted score.

## Materials and methods

### Sensors

For all data collections two IMUs mounted on both ski boots were used. The sensor setup and positioning was previously developed and validated for automatic ski turn detection [26] and ski style classification [27]. As in [26, 27] the configuration was 2.5 × 3 × 0.83 mm ±8 g ±500 dps full-scale, board by Movesense [28].

**Fig 1:**
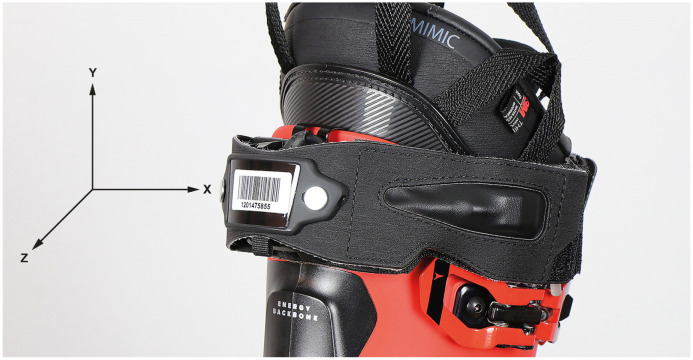
IMU attached to the ski boot via a strap.

The angular velocities of the accelerometer were recorded at 833 Hz. Analog and digital low-pass filters were applied directly by the IMU after A/D conversion and the signal was forwarded via Bluetooth at 54 Hz [28]. In order to ensure consistent data recording and exclude deviations in placement, the positioning and alignment of the sensors were kept the same for all participants. For all participants the IMUs were attached to the upper posterior cuff of the left and right ski boot, as illustrated in Fig 1. The IMU was fixed using a tight elastic strap and a customized rigid housing to avoid movement or misalignments. The X-axis corresponded to the lateral axis of the boot to the right, the Y-axis to the vertical axis of the boot upwards and the Z-axis was aligned according to the anterior-posterior direction. Apart from the IMUs mounted on the skiing boots, all jumps were additionally filmed with video recordings and a sampling rate of 25 Hz (Hero4 Session, GoPro, San Mateo, CA, USA [29]). Video and sensor data were synchronized using a jump event at the beginning and end of each trial, which is visible in the video and as a high peak of vertical acceleration in the IMU data of the boot. The synchronization jumps were detected in the vertical axis of the accelerometer and synchronized with the frame at landing.

### Data collection

**Fig 2:**
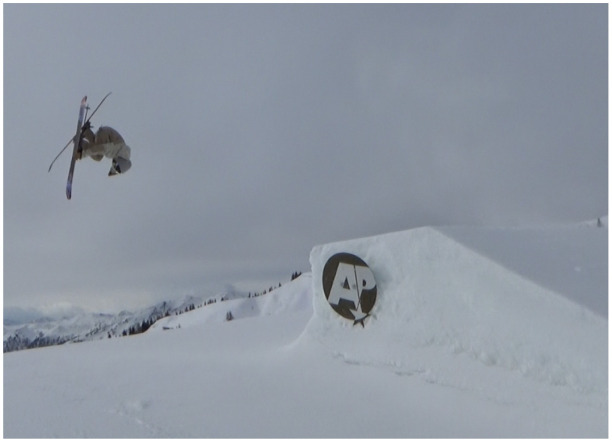
Example of a *Big Air jump*.

**Fig 3:**
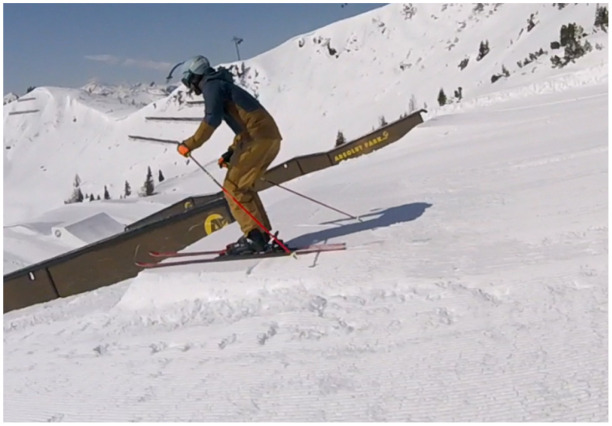
Example of a *jump during skiing*.

For the development and validation of the algorithm two types of jumps were performed. The recruitment period began on December 1, 2019 and lasted until December 31, 2019. All participants provided informed consent in written form. The study was approved by the local ethics committee of the University of Salzburg (GZ 11/2018). First, *Big Air jumps* were collected from four professional athletes who jumped over *Big Air jumps* in one day at a snowpark in an Austrian ski resort. To make the algorithm more robust to this type of jump, the athletes performed several types of flips and spins so the algorithm would be more challenged as shown in Fig 2. Secondly, *jumps during skiing* were collected from two recreational skiers who made jumps during conventional skiing, as shown in Fig 3. These jumps were performed on different days in the same Austrian ski resort. We divided *jumps during skiing* into *small* (flight time < 500 ms) and *medium* (flight time ≥ 500 ms) jumps. A total of 15 *Big Air*, 18 *small* and 34 *medium jumps* were collected.

### Algorithm and parameter settings

A simple structured and easy to implement algorithm was developed to detect the jumps based on the acceleration data from IMUs. The mean value of the absolute vertical accelerations (*Acc*_*y*_) of the left and right boot is used as initial data. Then there are two parameters, a rolling window to smooth the data and a value to which these smoothed values are rounded, which can be set to detect the respective jumps: First, the data is smoothed with a rolling window of *X* data points, where one data point corresponds to 18.5 ms for a collection rate of 54 hz. To find the best parameter settings, the rolling window is set between a value of 10–25 data points, which corresponds to a period from 185.2 ms to 463 ms. Second, these smoothed values are rounded to the nearest increments of five or ten (e.g. when rounded to the nearest five: a value of 3 is rounded to 5, a value of 23 is rounded to 25, etc.; when it is rounded to the nearest 10: a value of 3 is rounded to 0, a value of 23 is rounded to 20 etc.). Finally, the areas of zeros are identified as jumps. All calculations are made with the software R [30].

The following schematic representation of the algorithm should facilitate the understanding of its structure:

1. Taking the mean absolute values of the raw data: mean(abs($acc_{y_{left}}$), abs($acc_{y_{right}}$))
2. Smoothing this data with a rolling window: parameter setting of 10–25 data points used for the rolling window, corresponding to a period from 185.2 ms to 463 ms
3. Rounding these smoothed values to the nearest increments of five or ten
4. Identify the areas of zeros as jump region

**Fig 4:**
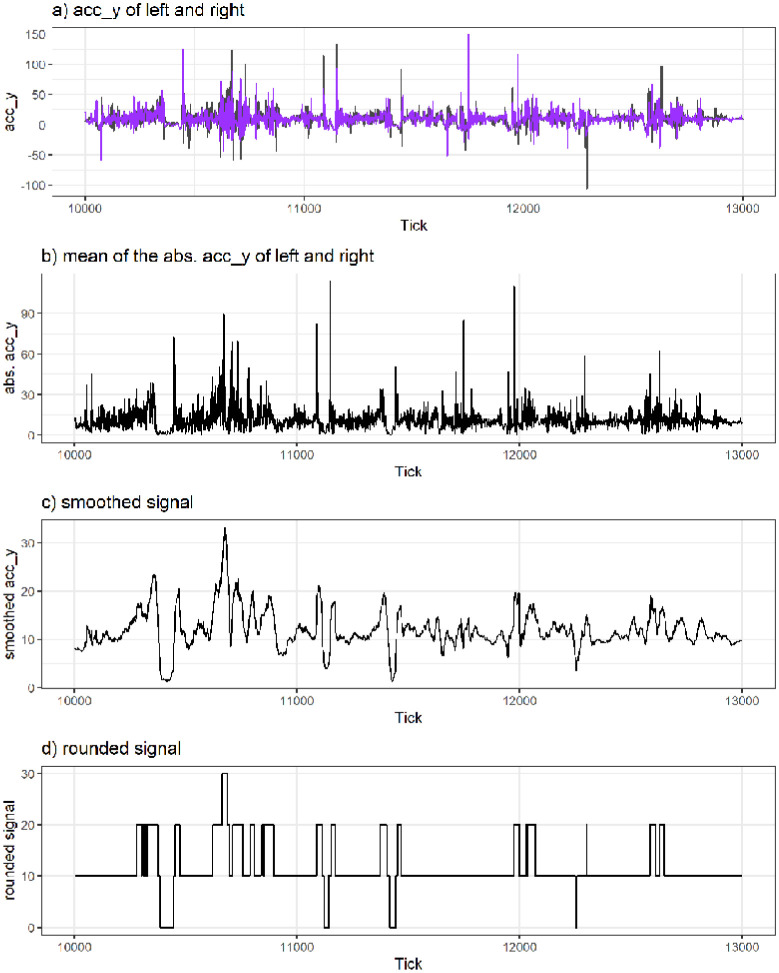
Example of IMU data processing steps for a rolling window of 463 ms and rounding to the nearest increment of 10.

Fig 4 visualises an example snapshot of the IMU data for *jumps during skiing*. Fig 4a) shows the raw signal of the left and right ski boot. Fig 4b) shows the mean value of the absolute vertical acceleration of the left and right ski boot. Fig 4c) shows the smoothed signal (based on a rolling window of 463 ms) and Fig 4d) illustrates rounding to the nearest increment of 10. A jump is detected when the rounded signal is equal to 0. In the case of Fig 4, four jumps were detected.

### Validation scores

To validate the algorithm, the jump start time detected in the video was compared with the jump start time calculated by the algorithm. For the jumps to match, the jump start times determined by the algorithm and the video recording must be within 150 ms. Otherwise, the jumps were considered undetected.

To evaluate the respective parameter settings, we present results for:

(1) the ratio of properly detected jumps over the total number of jumps and
(2) the number of overdetected jumps

for each combination of the two parameter settings (rolling window and value to which is rounded) respectively. For visualisation we used heat maps, which were created with the R package ggplot2 [31]. Moreover, we introduced a so-called penalty-adjusted score that takes into account the trade-off between (1) and (2). Thus, we first computed the (3) penalty score, which is the sum of the overdetected jumps divided by the total number of jumps (ground truth). In a next step, we subtracted the penalty score (3) from (1), the proportion of detected jumps to the total number of jumps. Consequently, the (4) penalty-adjusted score is lower when the number of overdetected jumps is high relative to the total number of jumps (ground truth).

$$\begin{array}{c} \text{relative}\;\text{ratio}\;\text{of}\;\text{detected}\;\text{jumps}=\frac{\text{number}\;\text{of}\;\text{detected}\;\text{jumps}}{\text{total}\;\text{number}\;\text{of}\;\text{jumps}\;(\text{ground}\;\text{truth})} \end{array}$$ (1)

$$\begin{array}{c} \text{number}\;\text{of}\;\text{overdetected}\;\text{jumps}=\sum \text{overdetected}\;\text{jumps}\;\text{per}\;\text{run} \end{array}$$ (2)

$$\begin{array}{c} \text{penalty}\;\text{score}=\frac{\text{number}\;\text{of}\;\text{overdetected}\;\text{jumps}}{\text{total}\;\text{number}\;\text{of}\;\text{jumps}\;(\text{ground}\;\text{truth})} \end{array}$$ (3)

$$\begin{array}{c} \text{penalty-adjusted}\;\text{score}=\text{relative}\;\text{ratio}\;\text{of}\;\text{detected}\;\text{jumps}-\text{penalty}\;\text{score} \end{array}$$ (4)

We have to note that it was not possible to calculate the number of overdetected jumps in *Big Air jumps*, since only one *Big Air jump* per ride was analyzed.

## Results

### Relative ratio of detected jumps

**Fig 5:**
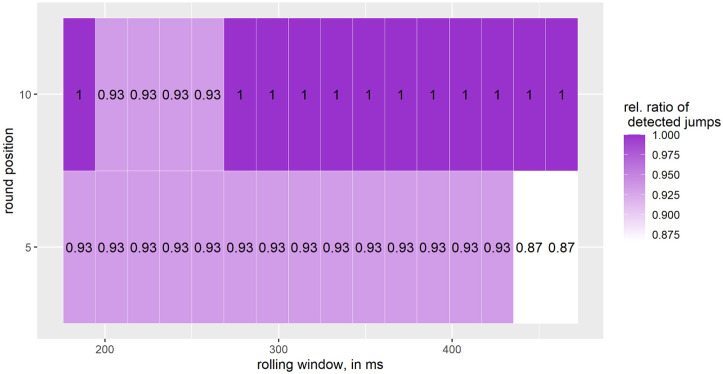
Relative ratio of detected *Big Air jumps*; number of Big Air jumps: 15; one interval on the X-axis corresponds to 18.5 ms starting at 185.2 ms and ranging to 463 ms.

Fig 5 shows that the detection rates for *Big Air jumps* range from 87% to 100%. The lowest detection rates are found for a rolling window between 444.4 − 463 ms and when rounding to the nearest 5, while the highest detection rates are found when rounding to the nearest 10 and a rolling window between 277.8 − 463 ms.

**Fig 6:**
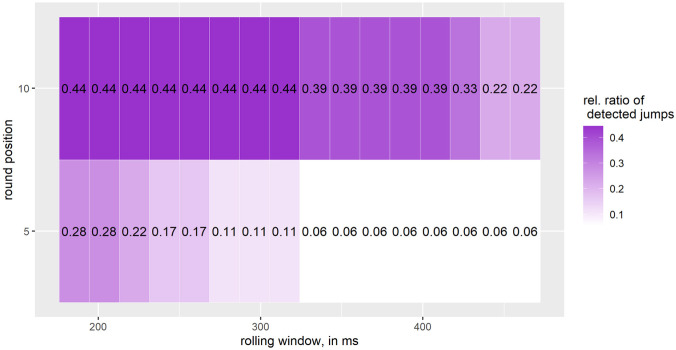
Relative ratio of detected *small jumps* (flight time < 500); number of small jumps: 18; one interval on the X-axis corresponds to 18.5 ms, starting at 185.2 ms and going up to 463 ms.

Fig 6 shows that for *small jumps* we find the highest relative ratio of detected jumps (44%) for a rolling window between 185.2–314.8 ms and when the IMU data is rounded to the nearest increment of 10. For a rolling window between 333.3 − 463 ms and rounding to the nearest 5, we have the lowest relative ratio of detected *small jumps*.

**Fig 7:**
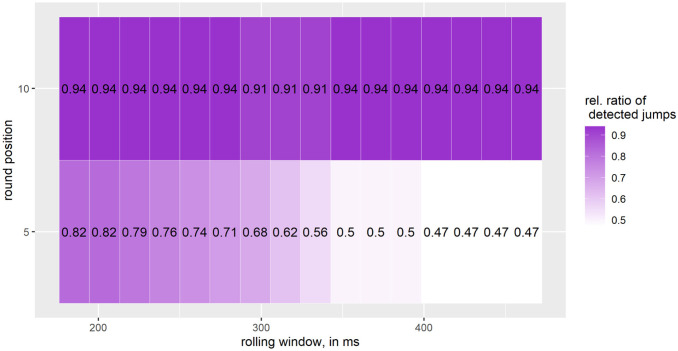
Relative ratio of detected *medium jumps* (flight time ≥ 500) jumps; number of medium jumps: 34; one interval on the X-axis corresponds to 18.5 ms, starting at 185.2 ms and going up to 463 ms.

For *medium jumps*, Fig 7 shows the highest detection rate of 94% for a rolling window between 185.2–277.8 or 351.8 − 463 ms and when the IMU data is rounded to the nearest 10. The lowest detection rate is for a rolling window between 407.4 − 463 and when rounded to the nearest 5.

**Fig 8:**
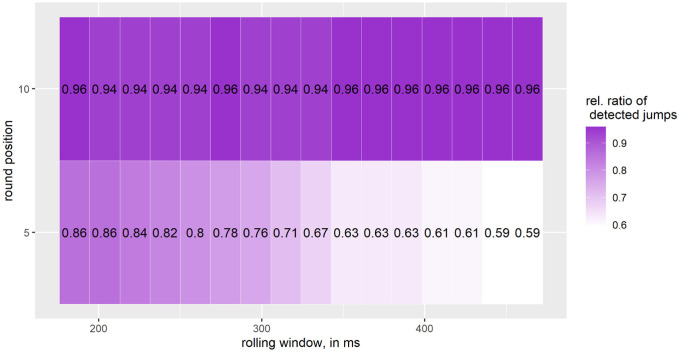
Relative ratio of detected *Big Air and medium jumps*; number of jumps: 49; one interval on the X-axis corresponds to 18.5 ms, starting at 185.2 ms and going up to 463 ms.

Fig 8 shows the results for all jumps with a flight time ≥ 500 ms, considering both *Big Air* and *medium jumps*. We find the highest detection rate for a rolling window of 185.2, 277.8 and between 351.8 − 463 ms and when rounding to the nearest 10. On the other hand, we find the lowest detection rate for a rolling window between 444.4 − 463 and when rounding to the nearest 5.

### Number of overdetected jumps and adjusted-penalty score

**Fig 9:**
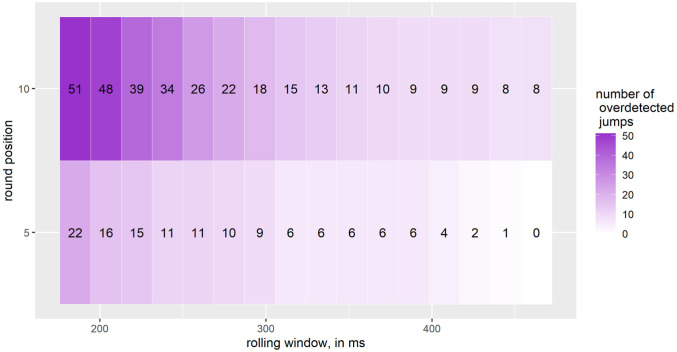
Number of overdetected jumps during skiing; small and medium jumps; number of jumps: 52; one interval on the X-axis corresponds to 18.5 ms, starting at 185.2 ms and going up to 463 ms.

Fig 9 shows that the smaller the rolling window, the higher the number of overdetected jumps for each rounding parameter. The results range from 51 to eight overdetected jumps when rounding to the nearest 10 and 22 to zero overdetected jumps when rounding to the nearest 5.

**Fig 10:**
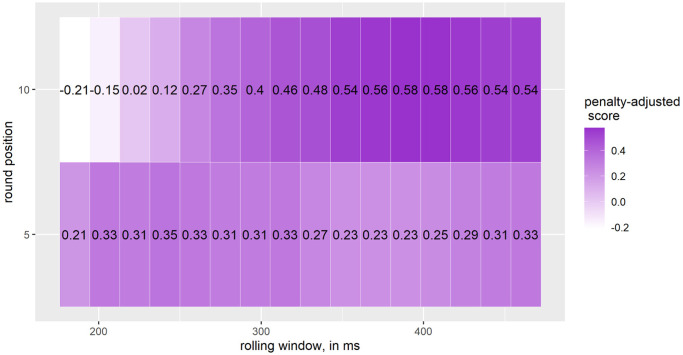
Penalty-adjusted score for jumps during skiing; small and medium jumps; number of jumps: 52.

Fig 10 shows the lowest and highest scores of all parameter combinations when rounding to the nearest 10, increasing from −0.21 to 0.58 between a rolling window of 185.2 to 407.4 ms. When rounding to the nearest 5, the penalty-adjusted score varies from 0.21 for a rolling window of 185.2 ms to 0.35 for a rolling window of 240.7 ms.

## Discussion and conclusion

The aim of this work was to develop a simple and easy-to-implement algorithm based on IMU acceleration data to detect jumps under different skiing conditions. The results showed a very high ratio of jumps detected with barely any overdetections for *small and medium jumps*.

For *jumps during skiing*, the algorithm has the highest detection rates for the smallest rolling window of 185.2 ms and when rounding to the nearest 10 for *small and medium jumps*, respectively. The lowest detection rates are found for a rolling window of 463 ms and when rounding to the nearest 5. Interestingly, the opposite picture is seen for the number of overdetected jumps: the highest number is found when rounding to the nearest 10 and using a rolling window of 185.2 ms, while we have no overdetected jumps when rounding to the nearest 5 and using a rolling window of 463 ms. Thus, there is a trade-off between the detection rate and the number of overdetected jumps. We therefore introduced what we call a penalty-adjusted score to account for this trade-off. According to the adjusted-penalty score, the best parameter settings are a rolling window of 388.9 or 407.4 ms and rounding to the nearest 10. Interestingly, the adjusted-penalty score increases for a rolling window of 185.2 to 388.9 ms, but decreases for a rolling window of 425.9 to 463 ms.

Compared to the results of Sadi and Klukas [22], which achieved a detection rate of 92% for jumps during snowboarding, we achieve slightly better results with a detection rate of 96%, for jumps with a flight time ≥ 500 ms, considering both *Big Air* and *medium jumps*. Compared to Roberts-Thomson et al. [24], which achieved a detection rate of 100% for ski jumping, the algorithm shows an equivalent detection rate of 100% with respect to *Big Air jumps*. However, the algorithm can be improved for jumps smaller than 500 ms, where it achieved a detection rate of 44%. The relatively low detection rate is likely due to the short flight time associated with a low vertical acceleration level and maybe some noise in the signal due to takeoff and landing accelerations.

The small number of participants who performed jumps is one of the limitations of the present study. To account for it, several different Big Air jump styles were performed, and different skill level skiers were included (professional and recreational). Furthermore, the algorithm is based on the detection of airtime, when the vertical acceleration is constant and the external vibrations are typically low, limiting the influence of anthropometrics and skill level. Nevertheless, a larger number and diversity of participants and jump characteristics could increase the validity and robustness of the algorithm.

Moreover, future studies should analyse multiple *Big Air jumps* during a run to calculate the number of overdetected jumps and the penalty-adjusted score.

In summary, the proposed algorithm works well for *medium jumps* but needs to be strengthened for *small jumps* with a time of flight less than 500 ms. For *Big Air jumps* all jumps could be detected, while 94% of all jumps with a flight time ≥ 500 ms could be detected during conventional skiing. The advantage of the proposed algorithm is that it provides an easy-to-implement structure, provides fast feedback, and has high accuracy for jump durations greater than 500 ms. Future work could further develop the algorithm to detect additional events, such as jump duration, and to classify the type of jump.
